# Mental health apps in India: regulatory landscape and future directions

**DOI:** 10.1192/bji.2024.20

**Published:** 2025-02

**Authors:** M. I. Singh Sethi, Rakesh C. Kumar, Narayana Manjunatha, Channaveerachari Naveen Kumar, Suresh Bada Math

**Affiliations:** 1 Senior Resident, Tele Medicine Centre, NIMHANS Digital Academy, Department of Psychiatry, National Institute of Mental Health & Neurosciences (NIMHANS), Bangalore, India; 2 Assistant Professor, Tele Medicine Centre, NIMHANS Digital Academy, Department of Psychiatry, National Institute of Mental Health & Neurosciences (NIMHANS), Bangalore, India; 3 Additional Professor, Tele Medicine Centre, NIMHANS Digital Academy, Department of Psychiatry, National Institute of Mental Health & Neurosciences (NIMHANS), Bangalore, India; 4 Head of Community Psychiatry, Department of Psychiatry, National Institute of Mental Health & Neurosciences (NIMHANS), Bangalore, India; 5 Head of Forensic Psychiatry & Tele Medicine Centre, I/C NIMHANS Digital Academy, Department of Psychiatry, National Institute of Mental Health & Neurosciences (NIMHANS), Bangalore, India.

**Keywords:** Mental health apps (MHAs), regulatory framework, digital health, data privacy, mobile health (mHealth)

## Abstract

Mental health apps (MHAs) are increasingly popular in India due to rising mental health awareness and app accessibility. Despite their benefits, like mood tracking, sleep tools and virtual therapy, MHAs lack regulatory oversight. India's framework, including the Central Drugs Standard Control Organization (CDSCO) and Medical Device Rules 2017, does not cover standalone health apps, raising concerns about data privacy and accuracy. Establishing a centralised regulatory body with guidelines for MHAs is essential for user safety and efficacy. This paper examines the current regulatory landscape, compares international approaches and proposes a tiered regulatory framework to foster responsible innovation while safeguarding user interests in digital mental health services.

Mental health apps (MHA) have surged in popularity in India due to the rising prevalence of mental health concerns, increased emphasis on mental well-being and widespread smartphone and internet access. These apps cater to various needs, from stress reduction to treatment for anxiety, depression and PTSD. They offer features like mood tracking, sleep tools and virtual therapy sessions. While MHAs can complement traditional therapy, they are not substitutes for professional care. Users need to exercise caution and consider the evidence behind each app they choose.^1^ This paper aims to examine India's current regulatory landscape for MHAs, compare it with international approaches and propose a tiered regulatory framework. We will analyse the existing regulations and their challenges, categorise different types of MHAs and their regulatory needs and recommend steps to establish a centralised regulatory body. By addressing these objectives, we seek to provide a comprehensive analysis of MHA regulation in India and offer concrete suggestions for improvement. The paper will discuss the current regulatory situation, analyse various MHA types, compare international approaches, propose a new framework and conclude with implications for future regulation.

## Current regulatory landscape and challenges

### Navigating the murky waters: challenges of app review and regulation

The Central Drugs Standard Control Organization (CDSCO), under the Ministry of Health and Family Welfare, is the primary regulatory body for medical devices in India. The Medical Device Rules 2017 classify medical devices into different categories based on their risk levels and regulate them accordingly.^2^ Software integral to the functioning of a medical device falls under this regulatory framework. This includes software used for diagnostics, monitoring or therapeutic purposes, which must meet specific safety, performance and quality standards. At present, standalone software applications not associated with any physical medical device are not regulated by the CDSCO. These standalone apps include health and wellness apps, mobile applications that track fitness or general health metrics and other software that monitors health status and illness through both active and passive digital phenotype tracking. There is no regulation on these standalone health or mental health apps.

While reviews are essential to assess the effectiveness, user satisfaction and adherence to ethical guidelines of MHAs, navigating India's evolving regulatory landscape adds complexity for users and developers alike. The effectiveness of these apps in addressing mental health issues has not been extensively researched.^3^ There is limited information on quality assurance and validation processes. Unlike medical devices and pharmaceutical products, which undergo rigorous evaluations by regulatory bodies, health apps often do not face such stringent requirements, raising concerns about data privacy violations, inaccurate information and unverified therapeutic claims.^4^

India lacks a centralised regulatory body to oversee MHAs, ensuring their safety, efficacy and adherence to ethical standards. Without such a body, consumers are left to navigate the vast landscape of available apps on their own, without clear guidance or assurance of their effectiveness or safety. Furthermore, this shifts the onus onto mental health professionals (MHPs) to independently assess the suitability of these apps and recommend them to their clients or patients.^5^ Table 1 depicts MHA regulations in certain countries. Similar comparison can be drawn to frame regulation in India using existing laws related to mental health in the country.^6,7^

**Table 1:** Comparison of mental health app (MHA) regulation across countries

Regulatory aspect	USA	UK	Australia	Japan	Germany	Singapore
Regulatory bodies	Primary: FDA, primarily via their Center for Devices and Radiological Health. Others: FTC, Department of HHS.	Primary: MHRA. Others: ORCHA, NHS, NICE.	Primary: TGA. Others: AHPRA, RANZCP.	PMDA.	Primary: BfArM. Others: hih, a think-tank by the German Federal Ministry of Health.	HSA.
Classification of apps	Risk-based (class I, II, III), ‘wellness’ versus ‘medical’ device distinction with less focus on tech type.	Risk-based, ‘wellness’ versus ‘treatment’ with some focus on tech (artificial intelligence guidance emerging).	Risk-based, ‘wellness’ versus ‘medical’ device distinction.	Risk-based, with SaMD guidance.	Risk-based, follows EU MDR.	Risk-based, follows HSA's risk classification system.
Evaluation standards	Evidence: varies by risk, may include clinical trials. Data security: strong focus on HIPAA compliance. Clinical oversight: increasingly important. Safety: crisis protocols, risk assessment. Transparency: clear labelling.	Evidence: ORCHA reviews, NICE guidance. Data security: GDPR standards. Clinical oversight: varies by app risk. Safety: focus on risk management. Transparency: clear labelling, potential for app store role.	Evidence: clinical validation guidelines. Data security: GDPR standards. Clinical oversight: RANZCP recommendations emphasise professional judgment. Safety: emphasis on risk mitigation. Transparency: potential for labelling.	Evidence: emphasis on quality management systems and clinical evidence. Data security: PMD Act and APPI. Clinical oversight: Varies by app risk. Safety: focus on risk management. Transparency: clear labelling.	Evidence: strict adherence to EU MDR standards, including clinical evaluation. Data security: GDPR standards. Clinical oversight: varies by app risk. Safety: focus on risk management. Transparency: clear labelling.	Evidence: focus on quality, safety, and efficacy. Data security: follows PDPA. Clinical oversight: varies by app risk. Safety: focus on risk management. Transparency: clear labelling.
Enforcement mechanisms	FDA: warning letters, recalls, market removal. FTC: actions against deceptive marketing.	MHRA: can enforce medical device regulations. ORCHA: primarily through influence on app stores.	TGA: regulatory enforcement powers. Limited information on app-specific enforcement.	PMDA: authority to conduct inspections and issue recalls.	BfArM: can issue warnings, recalls and market removal.	HSA: can issue warnings, recalls, and prosecute non-compliant manufacturers.
App store role	Varies by app store.	Potential for increasing role in regulation.	Potential for increasing role in regulation.	Limited direct involvement – relies on developer compliance.	Collaborates with EU-wide initiatives for app assessment.	Potential for future collaboration with government health app library.
Guidance for developers	FDA provides guidance documents and resources on medical device regulations, including software.	MHRA offers guidance on medical device regulations, including software.	TGA provides guidance on medical device regulations, including software.	PMDA provides guidance on SaMD development and regulation.	BfArM offers extensive guidance on MDR compliance for digital health apps.	HSA provides guidelines on mobile medical apps and telehealth products.
Post-market surveillance	FDA requires post-market surveillance for higher-risk devices.	MHRA requires post-market surveillance for medical devices.	Limited information available.	Required for higher-risk apps; manufacturers must report adverse events.	Rigorous post-market surveillance required under MDR.	Post-market surveillance required, and severity of requirements based on risk classification.
Public awareness	FTC enforces truth in advertising laws for health claims.	NHS provides information on approved MHAs.	Initiatives like Beyond Blue offer resources and support for mental health issues. Government programs also promote MHAs.	Government initiatives to promote proper use of health apps.	DiGA provides public information on approved apps.	The Health Promotion Board runs various campaigns such as ‘It's OKAY to Reach Out’ to improve mental health literacy and reduce stigma.

FDA, Food and Drug Administration; FTC, Federal Trade Commission; HHS, Health and Human Services; HIPAA, Health Insurance Portability and Accountability Act of 1996; MHRA, Medicines and Healthcare products Regulatory Agency; ORCHA, Organisation for the Review of Care and Health Applications; NHS, National Health Service; NICE, National Institute for Health and Care Excellence; GDPR, General Data Protection Regulation; TGA, Therapeutic Goods Administration; AHPRA, Australian Health Practitioner Regulation Agency; RANZCP, Royal Australian and New Zealand College of Psychiatrists; PMDA, Pharmaceuticals and Medical Devices Agency; SaMD, specific software as medical device; PMD, Pharmaceuticals and Medical Devices; APPI, Act on the Protection of Personal Information; BfArM, Bundesinstitut für Arzneimittel und Medizinprodukte [Federal Institute for Drugs and Medical Devices]; hih, health innovation hub; EU MDR, European Union Medical Device Regulation; DiGA, Digitale Gesundheitsanwendungen [Digital Health Apps Directory]; HSA, Health Sciences Authority; PDPA, Personal Data Protection Act.

### Legislations overlaying the evolving mental health service in India: patchwork to regulations

Major mental health legislations in India focus on ethics, registration and recognition of mental health establishments (MHEs) and MHPs. While MHAs cater to both general and clinical populations, current laws mainly cover conventional in-person services. The Mental Healthcare Act (MHCA) 2017 emphasises patient rights, professional qualifications and MHEs’ and MHPs’ registration. However, its applicability to MHAs is limited, necessitating specific regulations for these apps.^8^

Recent guidelines, such as India's Telemedicine Practice Guidelines (TPG) 2020 and Telepsychiatry Operational Guidelines, recognise telepsychiatry and address issues of informed consent, e-prescriptions and data security. Additionally, the Information Technology Act 2000, Section 43A, mandates ‘reasonable security practices’ for entities handling sensitive personal data and imposes penalties for wrongful loss or gain caused by negligence.^9^

MHAs, dealing with highly sensitive data, would need to adhere to these standards. The National Medical Commission (NMC's) Code of Medical Ethics^10^ underscores the importance of patient privacy as well. The lack of specificity related to MHAs reveals the need for a regulatory framework that builds upon the NMC's ethical principles. The proposed Data Protection Data Privacy Act (DPDP)^12^ also emphasises patient control over health data, including rights to access, correct and potentially erase data, along with the ability to restrict its use without explicit consent, object to automated decision-making and file complaints about violations. However, on the other hand, the act doesn't restrict health data transfer when the data are shared outside of India. Though this enables international research collaborations or use of foreign-based cloud services, it raises alarm on inviting further debate on legal jurisdictions across borders.^11^

Providing a broader purview, the Drugs, Medical Devices, and Cosmetics Bill (DMDCB) 2022 defines ‘medical device’ as that which could encompass MHAs functioning on a diagnostic or therapeutic capacity.^12^ Additionally, the DMDCB emphasises evidence-based validation for therapeutic claims^12^). The Indian Council of Medical Research (ICMR) has established guidelines for the ethical use of artificial intelligence in healthcare. These guidelines are particularly important for developers of MHAs powered by artificial intelligence. The ICMR also emphasises patient safety, data privacy, transparency and the mitigation of bias in artificial intelligence algorithms. Observing the trend, it is understood that the country is moving towards regulating laws and guidelines in mental health, which provides more clarity on MHAs’ role, their utility and efficacy in interventions, diagnosis and treatment.^13^ The requirement is currently only a patchwork overlaying these above-mentioned legislations.

### Types of MHAs and regulatory needs

MHAs encompass a diverse range of tools with varying purposes and regulatory implications. These apps range from wellness and self-help tools requiring minimal oversight to artificial intelligence-powered therapeutic platforms demanding rigorous regulation. Wellness apps focus on general mental well-being, while symptom-tracking and mood apps necessitate moderate regulation to ensure data security and algorithm accuracy.

Psychoeducation apps provide mental health information, requiring verification of content accuracy. Therapy support apps complement professional care, demanding integration with healthcare systems. Digital therapeutics (DTx) deliver evidence-based interventions, necessitating clinical trials and potential classification as medical devices. Teletherapy platforms connect users with professionals, requiring stringent regulation of provider credentials and crisis management protocols.

The most complex category, artificial intelligence-powered therapeutic apps, requires the highest level of regulation, including extensive testing and ongoing monitoring. This diverse landscape, as detailed in Table 2, underscores the need for a tiered regulatory approach in India, where oversight is calibrated to the potential risks and benefits associated with each type of MHA.^14^

**Table 2:** Types of mental health apps and regulatory needs

Category	Subcategory	Data handling	Regulatory needs
1. Wellness apps	Meditation and mindfulness	User-generated	Low: focus on data privacy, accurate marketing.
	Stress management	User-generated	Low to moderate: efficacy claims verification.
	Sleep improvement	User-generated, Device data	Moderate: accuracy of tracking, advice quality.
2. Illness apps	Symptom tracking	User-generated, PHI	High: data security, algorithm accuracy.
	Treatment seeking	User-generated, PHI	Very high: proper referral, crisis management.
	Relapse prevention	User-generated, PHI	Very high: efficacy validation, care integration.
	Digital therapeutics	User-generated, PHI	Extremely high; clinical trials, medical device classification.
3. Combination apps	Comprehensive artificial intelligence platforms	Mixed	Extremely high: feature-dependent regulation, algorithm accuracy, data security, validation.
	Teletherapy + self-help	User-generated, PHI	Extremely high: provider credentials, data security, efficacy.

PHI, Patient Health Information.

## Implications and conclusions

To safeguard users’ interests and promote responsible app development, it is high time that a centralised regulatory body could establish guidelines and standards for MHAs in the country. This would create a more consistent and robust approach for authentication which should include verifying the credibility and qualifications of app developers, ensuring evidence-based content and practices, compliance with ethical standards and scrutinizing data security measures. India can also draw valuable insights from the international frameworks (Table 1), where only higher-risk mental health treatment apps undergo strict medical device regulation by working alongside local expert organisations like the Indian Psychiatric Society and technology and legal agencies. Additionally, the regulatory body should offer support to smaller developers and establish mechanisms for user feedback. By implementing these recommendations, India can become a leader in responsible MHA regulation, safeguarding users, promoting innovation, and ultimately improving access to effective digital mental health services.

## Data Availability

Data availability is not applicable to this article as no new data were created or analysed in this study.

## References

[1] Singh S, Sagar R. Time to have effective regulation of the mental health apps market: maximize gains and minimize harms. Indian J Psychol Med 2022; 44(4): 399–404. 35949633 10.1177/02537176221082902 PMC9301749

[2] Manu M, Anand G. A review of medical device regulations in India, comparison with European Union and way-ahead. Perspect Clin Res 2022; 13(1): 3–11. 35198422 10.4103/picr.PICR_222_20 PMC8815674

[3] Johnson JA, Sanghvi P, Mehrotra S. Technology-based interventions to improve help-seeking for mental health concerns: a systematic review. Indian J Psychol Med 2022; 44(4): 332–40. 35949632 10.1177/02537176211034578 PMC9301737

[4] Sinha Deb K, Tuli A, Sood M, Chadda R, Verma R, Kumar S, et al Is India ready for mental health apps (MHApps)? A quantitative-qualitative exploration of caregivers’ perspective on smartphone-based solutions for managing severe mental illnesses in low resource settings. PLoS One 2018; 13(9): e0203353. 30231056 10.1371/journal.pone.0203353 PMC6145572

[5] Menon V, Rajan TM, Sarkar S. Psychotherapeutic applications of mobile phone-based technologies: a systematic review of current research and trends. Indian J Psychol Med 2017; 39(1): 4–11. 28250552 10.4103/0253-7176.198956 PMC5329989

[6] Powell A, Singh P, Torous J. The complexity of mental health app privacy policies: a potential barrier to privacy. JMIR mHealth uHealth 2018; 6(7): e9871. 10.2196/mhealth.9871 PMC6090172 30061090

[7] Essén A, Stern AD, Haase CB, Car J, Greaves F, Paparova D, et al Health app policy: international comparison of nine countries’ approaches. npj Digit Med 2022; 5(1): 31. 35304561 10.1038/s41746-022-00573-1 PMC8933556

[8] Math SB, Basavaraju V, Harihara SN, Gowda GS, Manjunatha N, Kumar CN, et al Mental Healthcare Act 2017 – aspiration to action. Indian J Psychiatry 2019; 61(Suppl 4): S660–6. 31040454 10.4103/psychiatry.IndianJPsychiatry_91_19 PMC6482691

[9] Sharma P, Sethi MIS, Liem A, Bhatti HBS, Pandey V, Nair A. A review of telemedicine guidelines in the South-East Asia region. Telemed Rep 2023; 4(1): 271–8. 37753247 10.1089/tmr.2023.0040 PMC10518689

[10] National Medical Commission. Code of Medical Ethics Regulations, Ch. 2, section 2.2. National Medical Commission, 2002 (https://www.nmc.org.in/rules-regulations/code-of-medical-ethics-regulations-2002/).

[11] Centre for Innovation, Intellectual Property and Competition. Indian Competition & Regulation Report 2023. Centre for Innovation, Intellectual Property and Competition, 2023 (https://circ.in/pdf/Report-ICRR2023.pdf).

[12] Naithani P. Protecting healthcare privacy: analysis of data protection developments in India. Indian J Med Ethics 2023; IX(2): 149. 10.20529/IJME.2023.078 38755773

[13] Sethi MI, Kumar CN, Math SB. The vanguard of psychiatry: artificial intelligence as a catalyst for change. J Psychiatry Spectr 2024; 3(1): 1–3.

[14] Lagan S, Aquino P, Emerson MR, Fortuna K, Walker R, Torous J. Actionable health app evaluation: translating expert frameworks for evaluation into a tool for health app users. npj Digit Med 2020; 3(1): 100. 32821855 10.1038/s41746-020-00312-4 PMC7393366

